# Contribution of fish farming ponds to the production of immature *Anopheles* spp. in a malaria-endemic Amazonian town

**DOI:** 10.1186/s12936-015-0947-1

**Published:** 2015-11-14

**Authors:** Izabel Cristina dos Reis, Cláudia Torres Codeço, Carolin Marlen Degener, Erlei Cassiano Keppeler, Mauro Menezes Muniz, Francisco Geovane Silva de Oliveira, José Joaquin Carvajal Cortês, Antônio de Freitas Monteiro, Carlos Antônio Albano de Souza, Fernanda Christina Morone Rodrigues, Genilson Rodrigues Maia, Nildimar Alves Honório

**Affiliations:** Programa de Computação Científica-PROCC, Fundação Oswaldo Cruz, Rio de Janeiro, RJ Brazil; Laboratório de Transmissores de Hematozoários, Instituto Oswaldo Cruz (Fiocruz), Rio de Janeiro, RJ Brazil; Núcleo Operacional Sentinela de Mosquitos Vetores, NOSMOVE (Parceria DIRAC-IOC-VPAAPS/FIOCRUZ), Rio de Janeiro, RJ Brazil; Centro Multidisciplinar, Universidade Federal do Acre, Cruzeiro do Sul, Acre Brazil; Laboratório de Doenças Parasitárias, Instituto Oswaldo Cruz (Fiocruz), Rio de Janeiro, RJ Brazil; Secretaria Municipal de Saúde de Cruzeiro do Sul, Cruzeiro do Sul, Acre Brazil; Secretaria de Estado de Agropecuária de, Cruzeiro do Sul, Acre Brazil

**Keywords:** *Anopheles* spp., *Anopheles darlingi*, Fishponds, Natural mosquito breeding habitat, Malaria

## Abstract

**Background:**

In the past decade fish farming has become an important economic activity in the Occidental Brazilian Amazon, where the number of new fish farms is rapidly increasing. One of the primary concerns with this phenomenon is the contribution of fishponds to the maintenance and increase of the anopheline mosquito population, and the subsequent increase in human malaria burden. This study reports the results of a 2-year anopheline abundance survey in fishponds and natural water bodies in a malaria-endemic area in northwest Brazil. The objective of this study was to investigate the contribution of natural water bodies (rivers, streams, creeks, ponds, and puddles) and artificial fishponds as breeding sites for *Anopheles* spp. in Mâncio Lima, Acre and to investigate the effect of limnological and environmental variables on *Anopheles* spp. larval abundance.

**Methods:**

Natural water bodies and fishponds were sampled at eight different times over 2 years (early, mid and late rainy season, dry season) in the Amazonian town of Mâncio Lima, Acre. Anopheline larvae were collected with an entomological dipper, and physical, chemical and ecological characteristics of each water body were measured. Management practices of fishpond owners were ascertained with a systematic questionnaire.

**Results:**

Fishponds were four times more infested with anopheline larvae than natural water bodies. Electrical conductivity and the distance to the nearest house were both significant inverse predictors of larval abundance in natural water bodies. The density of larvae in fishponds raised with increasing border vegetation. Fishponds owned by different farmers varied in the extent of anopheline larval infestation but ponds owned by the same individual had similar infestation patterns over time. Commercial fishponds were 1.7-times more infested with anopheline larvae compared to fishponds for family use.

**Conclusions:**

These results suggest that fishponds are important breeding sites for anopheline larvae, and that adequate management activities, such as removal of border vegetation could reduce the abundance of mosquito larvae, most importantly *Anopheles darlingi*.

## Background

Malaria, one of the most prevalent infectious diseases, is caused by parasites of the genus *Plasmodium* (Apicomplexa: Plasmodiidae) and is transmitted to humans via the bite of infected female *Anopheles* (Diptera: Culicidae) mosquitoes. *Anopheles darlingi* is a highly anthropophilic and efficient malaria vector that is widely prevalent in the Brazilian Amazon basin [1]. At least 33 other anopheline species exist in the Brazilian Amazon region [2, 3] and several of them, including *Anopheles deaneorum*, *Anopheles braziliensis*, *Anopheles nuneztovari*, *Anopheles oswaldoi s.l*, *Anopheles triannulatus*, *Anopheles strodei*, *Anopheles evansae*, *Anopheles galvaoi*, *Anopheles aquasalis*, *Anopheles albitarsiss.l*, and *Anopheles peryassui* have also been implicated as malaria vectors in the Amazon [3–6].

The wide diversity of neo-tropical anophelines has been attributed to the genus’ ability to adapt to numerous niches [7]. Anopheline larvae habitats range from fresh and salt-water marshes, to mangrove swamps, rice paddies, grass-filled ditches, the borders of rivers and streams, and small transient puddles of water [8, 9]. Environmental factors such as the physical and chemical characteristics of the water, as well as vegetation type, directly and indirectly influence anopheline ovipositing behaviour, larval distribution, population density, and development [10, 11]. The knowledge of factors that affect larval breeding sites is requisite to understand the space–time distribution of the mosquitoes and to develop appropriate vector-control strategies.

Fish farming activities are generally implemented in rural areas. The town of Mâncio Lima is an exception, as numerous fishponds were constructed in recent years. The town is located at the margin of an *igapó* forest (blackwater-flooded forest), which is seasonally flooded with fresh water, and crisscrossed by small streams. This provides ideal conditions for the easy construction of fishponds by either digging ponds beside rivers, or by damming them. In recent years, fish farming has blossomed, with roughly one fishpond for every 20 houses. The objective of this study was to investigate the contribution of natural water bodies (rivers, streams, creeks, ponds, and puddles) and artificial fishponds as breeding sites for *Anopheles* spp. in Mâncio Lima and to investigate the effect of limnological and environmental variables on *Anopheles* spp. larval abundance.

## Methods

### Study site

**Fig. 1:**
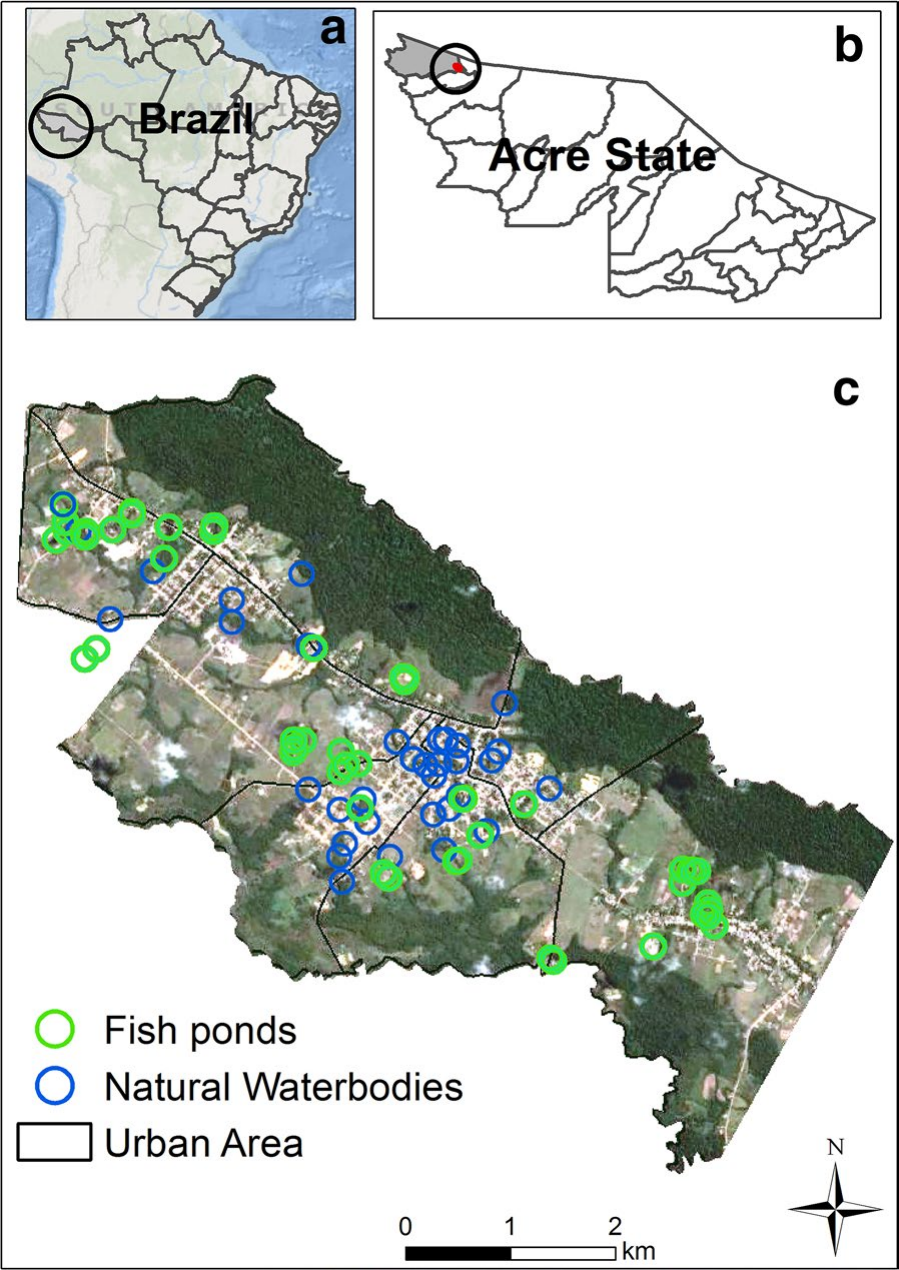
Maps of the study area. **a** Brazil and Acre State; **b** Acre State; *grey* highlights the municipality of Mâncio Lima while *red* denotes the location of the study site within Mâncio Lima; **c** Google Earth satellite map of the study area showing the location and types of water bodies where mosquito larvae were sampled

The study was performed in the town of Mâncio Lima (7°37′33.42″S, 72°53′29.89″W) in northwest Acre, Brazil (Fig. 1). The municipality’s 15,246 inhabitants (population density: 2.8 residents/km^2^) are spread through urban (57.6 %) and rural/riparian landscapes (42.4 %). The town is located on a mosaic of fragmented primary forest, animal pastures, man-made structures, and a diverse array of different water bodies (including the *Japiim* River, dams, streams, narrow channels with riparian morich palms, perennial and temporal puddles, and pisciculture-focused fishponds). It is divided into nine neighbourhoods. Downtown is the most urbanized, with paved streets and numerous commercial activities. The other neighbourhoods spread over a peri-urban landscape that consists mostly of unpaved roads, small farms, empty fields, and small clusters of homes. The major economic activities in the region are manioc flour production and pisciculture [12]. The hot and humid climate is characterized by rainy (April to November) and dry seasons (December to March) [13]. Average annual precipitation is 2,100 mm and the mean annual temperature is 25.5 °C (monthly minimum and maximum temperatures: 19–32 °C) [14].

Mâncio Lima is among the ten most malaria-affected municipalities in the Brazilian Amazon [15]. The number of notified malaria cases has increased considerably since 2004, coinciding with the expansion of fish farming activities in the area [16]. Investment in fish farming is an integral part of the Brazilian Federal Government’s poverty alleviation programme, which focuses on enhancing local economies [17, 18]. In 2006, a malaria epidemic occurred in Mâncio Lima with 16,125 cases [annual parasite index (API) = 1217.8] [19]. After enforcing mosquito control measures, the number of malaria cases declined to 4398 cases in 2008 (API = 305.9), but experienced a recent surge in 2014 with 6016 reported cases (API = 380.8).

Mâncio Lima is a major commercial access point for riverine and rural communities in the same municipality, and the neighbouring municipality of Rodrigues Alves. People frequently commute between rural areas and Mâncio Lima town to exchange commodities, procure medical attention, and access social welfare [12]. The high prevalence of malaria in these rural and riverine regions, and the frequent human traffic to and from Mâncio Lima, contributes to the town`s vulnerability to malaria epidemics.

### Mapping of water bodies

A satellite image (OpenStreetmap, October 12, 2011) served as a base to draw a street map and to locate fishponds. A field inspection was conducted in November 2011 to verify pond locations and use, and to identify additional fishponds with the help of residents. The study inclusion criterion was a distance of less than 2 km from the closest neighbourhood centre. When a property had more than three fishponds, up to three ponds were randomly chosen to be included in the survey.

Streams and wetlands were the predominant natural water bodies in the study area. A convenient sample of natural water bodies was identified, prioritizing those with relative proximity to streets and homes. All water bodies were geo-referenced with global positioning system (GPS).

### Study design

**Fig. 2:**
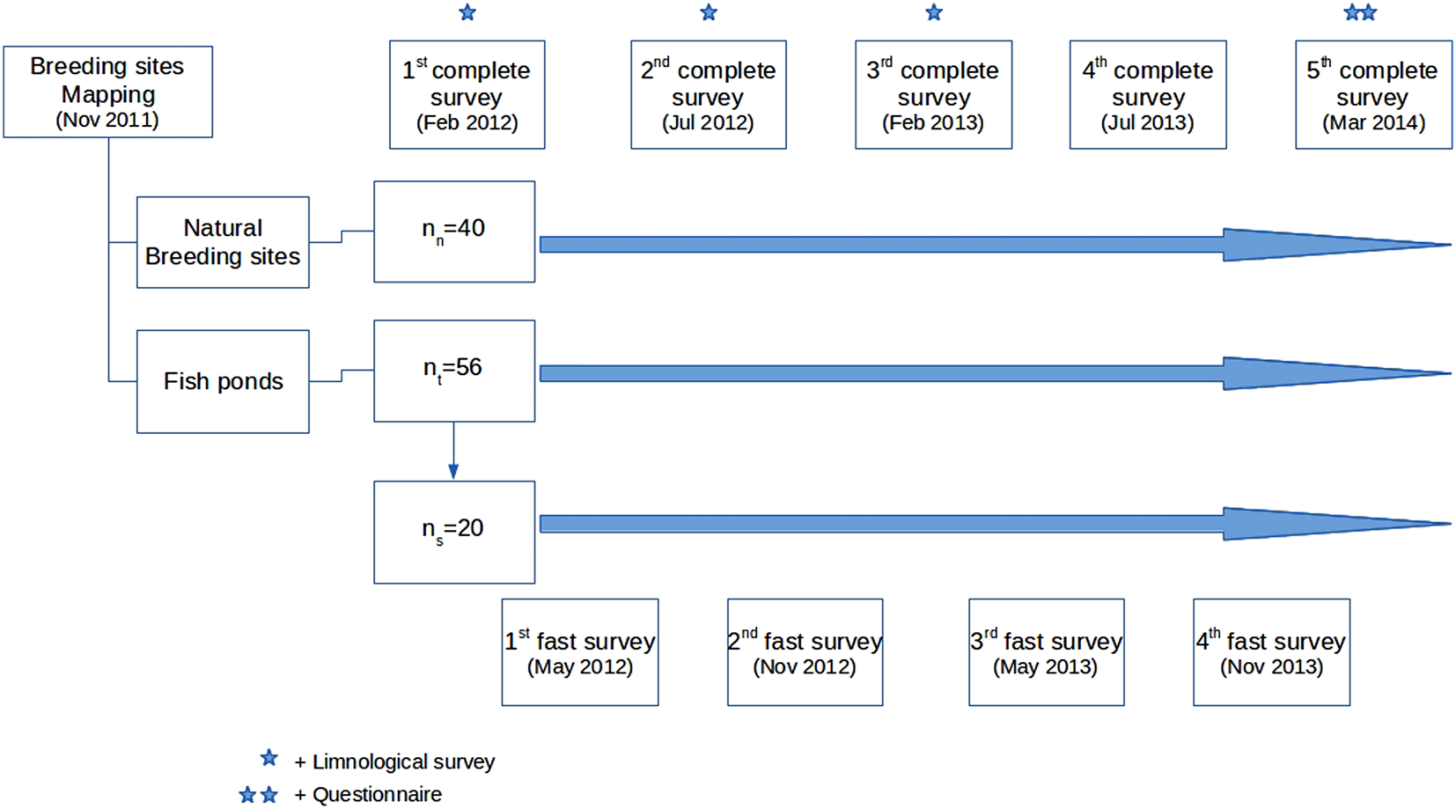
Sampling scheme of each of the nine mosquito larvae surveys of natural and artificial water bodies in Mâncio Lima between February 2012 and March 2014

The collection scheme is described in Fig. 1. All 55 fishponds and 21 natural water bodies were included in each of the five complete surveys. These were carried out approximately every 6 months between February 2012 and March 2014, once during each rainy and dry season. Nineteen of the 55 fishponds were also sampled in between these complete surveys (henceforth referred to as ‘fast surveys’). Collections for this sub-set occurred roughly every 3 months, encompassing the middle of the rainy season (February 2012 and 2013, March 2014), the middle of the dry season (July 2012 and 2013) and early and late rainy season, which are intermediate or transitional seasons (May 2012 and 2013, November 2012 and 2013) (Fig. 2). In this way, a dataset with greater temporal resolution for 19 (34.5 %) of the fishponds was obtained.

### Entomologic sampling

A standard 0.5 L dipper (Bioquip Co, Gardena, CA, USA) was used for sampling of immature *Anopheles* spp. as previously described [20]. The number of samples (dips) per water body varied from 20 to 155 depending on the length of the (accessible) border and size of the water body [21]. The number of *Anopheles* spp. larvae collected per dip per water body was recorded. Collected larvae were placed into Whirl–Pak^®^ sampling bags (Nasco Corp, Fort Atkinson, WI, USA; dimensions: 118 mL, 8 × 18 cm) half-filled with water from the collection site. The bags were sealed to retain air, placed in a container for transport, and transferred to the Núcleo Operacional Sentinela de Mosquitos Vetores (NOSMOVE/FIOCRUZ) in Rio de Janeiro. Third and fourth instar larvae were preserved in 70 % alcohol for identification. First and second instars were reared in plastic basins until reaching the third or fourth instar. All immature larvae were identified to species by Consoli and Lourenço-de-Oliveira [1].

The following descriptive ecological characteristics of each sampling area were recorded during each survey: type and size of water body, if flowing or standing water, type of usage, proportion of border with vegetation, and the presence or absence of macrophytes. The proportion of borders covered with vegetation was visually estimated. The distance (in m) between water bodies and the nearest human dwelling was measured with a flexible ruler.

### Limnological data

A multi-parameter water quality sonde (YSI Inc. 6600V2, Yellow Springs, OH, USA) was used to measure pH, temperature (°C), ammonium (mg/L), chlorophyll (mg/L), nitrate (mg/L), electrical conductivity (µS/cm), dissolved oxygen (mg/L), turbidity (NTU, nephelometric turbidity unit). Every water body was sampled twice (at its opposite ends) and the mean value of both samples was recorded. Limnological data only were collected during the first three complete surveys (February 2012, July 2012 and February 2013). These measurements were taken concomitantly to the mosquito larval sampling in each water body.

### Standardized questionnaire for owners of fishponds

The 55 surveyed fishponds belonged to 31 different owners, 30 of whom were interviewed during the final full sample conducted in February 2014. The questionnaire ascertained the following information: commercial fish farming or not, type of fish food, if the reared fish species was always the same, as well as if and how often each pond was emptied and refilled.

### Data analysis

The data were analysed in five steps. An overview is provided in Table 1. Generalized linear mixed models (GLMM) with a negative binomial distribution were used in all analyses because the variance of the number of larvae was greater than the mean, and because adjusted Poisson models were highly over-dispersed. Possible non-linear relationships between explanatory variables and the abundance of *Anopheles* spp. larvae were also considered by adjusting generalized additive mixed models (GAMM) with R’s ‘mgcv’ library [22] (results not shown because all co-variables were linear or approximately linear). All models included the water body ID code as a random effect to control for multiple samples of the same body of water over time. R version 3.1.2 [23] and the ‘MASS’ [24] and ‘lme4’ libraries [25] were used. All GLMM models had the following basic structure:

1 $$ \begin{aligned} & Anopheles_{ti} \sim {\text{ Negative binomial }}\left( {\mu_{ti} , \, k} \right) \\ & { \log }\left( {\mu_{ti} } \right) \, = { \log }\left( {N_{ti} } \right) + \beta {\text{X}}_{ti} + a_{i} \\ \end{aligned} $$

$$ {\text{a}}_{\text{i}} \sim {\text{ N}}\left( {0,\sigma_{\text{a}}^{ 2} } \right) $$

where log(*N*_*ti*_), the natural logarithm of the number of dips at time *t* in *water body i*, is the model offset to correct for the differences in the number of dips; *a*_*i*_ is the random intercept accounting for the repeated measures design; X represents explanatory variables (multiple covariates were adjusted in some cases); and β are the fixed effects of variables X.

**Table 1:** Overview of data analyses after mosquito larvae sampling in Mâncio Lima, 2012–2014

Analysis	Research question	Datasets
1	Is there a difference in *Anopheles* spp. larval infestation in natural water bodies and fishponds?	D5: All five complete surveys
2	Is *Anopheles* spp. larval infestation influenced by limnological and ecological covariates?	D3n: First three complete surveys, natural bodies of water sub-set D3f: First three complete surveys, fishpond sub-set
3	Does larval infestation change over time?	D5n: All five complete surveys, natural breeding site sub-set D9f: Five full and four short surveys, fishpond sub-set (only the 20 fishponds sampled during short and complete surveys)
4	Does larval infestation in fishponds vary by owner?	D5f: All five complete surveys, fishpond sub-set
5	Factors related to differences in larval infestation between owners?	D5f: All five complete surveys, fishpond sub-set

It was first investigated if ponds and natural breeding sites were similarly infested by *Anopheles* larvae. The dummy variable ‘type of water body’ (*type* = 0 or 1; fishponds or natural breeding site, respectively) was the primary effect. As larval infestation differed significantly between the two types of breeding sites and as some explanatory variables were only available for fishponds, the second step (investigation of the effect of limnological and ecological variables on larvae infestation) was carried out separately each of the two types of water bodies. Separate univariate GLMM models were adjusted for each variable. The AICs (Akaike information criterion) of models with significant effects (*p* value <0.1) were compared and a full model was adjusted. The co-variables of the full model were included in order of increasing AIC. Variables that were not statistically significant in the full model were removed in a stepwise fashion until the final model included only those that remained statistically significant (*p* < 0.05). In the case of collinearity, only one variable (lowest AIC in the univariate model) was included in the multiple model. The third step was an investigation of the influence of time on larval infestation. The main effects were either *collection* (collection = 1, …, 5 for natural breeding sites and collection = 1, …, 9 for fishponds), or *month* (month = 2, 7 for natural breeding sites and month = 2, 5, 7, 11 for fishponds). In the fourth analysis, the effect of the each pond’s *owner* (owner = 1, …, 30) on larval infestation in fishponds was evaluated. The owner ID code was included as a random effect in Eq. (1), instead of water body ID, and the resultant model was compared to the model without other covariates. The last step of the data analysis included an evaluation of the effect of variables from the questionnaire. The modelling strategy was the same as described in step 2. As only the variable *commercial* (*c* = 0 for commercial and *c* = 1 for non-commercial fishponds) had an effect on larval density, it was also evaluated if the proportion of *border vegetation*, which was highly significant in step 2, had different effects on commercial and non-commercial fishponds:

2 $$ \begin{aligned} & Anopheles_{ti} \sim {\text{ Negative binomial }}\left( {\mu_{ti} , \, k} \right) \\ & { \log }\left( {\mu_{ti} } \right) \, = { \log }\left( {N_{ti} } \right) + {\text{ f}}_{Ci} \left( v \right) + a_{i} \\ \end{aligned} $$

$$ {\text{a}}_{\text{i}} \sim {\text{ N}}(0,\sigma_{\text{a}}^{ 2} ) $$

where f_*Ci*_(*v*) is the smooth non-linear effect of *border vegetation v* in each water body *i* (i = 1, …, 56), among non-commercial and commercial fishponds. The other parameters are as previously described.

### Ethical considerations

The paper reports data from entomological surveys carried out using standard methods, in private and public spaces. Access to private spaces was requested to each land owner, and collections carried out only after their oral consent. Access to public spaces did not require permission but before taking place, the overall study was presented and approved by local Health and Environmental Secretariats. The only request was that the results were presented to the population, which occurred in the form of talks in the town’s conference room and in schools. The paper also reports data from interviews. A signed consent preceded the interviews. All measures were taken to guarantee confidentiality. The study was approved by the ethical committee at the Oswaldo Cruz Foundation in Rio de Janeiro (CEP Fiocruz n.402.039). Participants’ names were not recorded but instead, identification numbers of the fishponds were used. All the information was treated confidentially and only available to those directly concerned with this research.

## Results

### Physical characteristics of water bodies

Of the 93 monitored water bodies, 55 (59.1 %) were fishponds, 33 (35.5 %) flowing water (creeks, streams, and river) and five (5.4 %) small areas of standing water (ponds and puddles). Fishponds varied in size, with perimeters ranging from 24 to 900 m (average perimeter = 170.7 m). Most flowing waters (29, 89 %) were creeks, ranging from 0.5 to 50 m in width. The average distance between the potential breeding habitats and the closest human domicile was 37 m. Sixty-nine per cent of fishponds (n = 38) were used for commercial purposes.

### Anopheline abundance

A total of 21,156 *Anopheles* spp. larvae were collected (Table 2). Fifty-three per cent of the larvae were not identified because of larval mortality prior to third stage. The following eight anopheline species were identified among the 9944 (47 %) of the surviving and identifiable larvae: *An. albitarsiss.l*. (75.8 %), *An. darling* (16.1 %), *An. deaneorum* (6.1 %), *An. brasiliensis* (<1 %), *Anopheles argyritarsis* (<1 %), *An. triannulatus* (<1 %), *Anopheles rondoni* (<1 %), *An. galvaoi* (<1 %) (Table 2). Of all water bodies that were investigated during the five complete surveys (55 fishponds and 21 natural water bodies), 46 fishponds and eight natural water bodies were positive for *An. darlingi*. Twelve fishponds and seven natural water bodies were positive for *An. darlingi* once; five fishponds and one water body two times; 11 fishponds were positive at three different times; 12 fishponds four times; and six fishponds all five times they were sampled.

**Table 2:** Descriptive statistics of anopheline larvae from Mâncio Lima, Acre, Brazil collected between February 2012 and March 2014

	Fishponds			Natural water body		
	N (water bodies) = 337^a^, N (dips) = 23,085			N (water bodies) = 88^a^, N (dips) = 4456		
	Total	Mean larvae/dip	SD	Total	Mean larvae/dip	SD
*Anopheles* spp.	20,553	0.89	89.68	603	0.13	18.84
Not-identified	10,954	0.47	63.08	196	0.04	5.08
*An. darlingi*	1549	0.07	9.72	27	0.006	1.10
*An. deaneorum*	593	0.03	3.66	37	0.008	1.52
*An. albitarsis s.l*	7274	0.32	34.45	314	0.07	14.68
*An. triannulatus s.l*	94	0.004	0.89	8	0.001	0.47
*An. argyritarsis*	76	0.003	1.81	8	0.001	0.53
*An. braziliensis*	10	0.0004	0.12	7	0.001	0.74
*An. rondoni*	3	0.0001	0.26	6	0.001	0.63

^a^Adjusted for repeated samples over time

The mean number of anopheline larvae per dip (±standard deviation) in fishponds and natural water bodies were, respectively, 1.02 ± 1.77 and 0.20 ± 0.40. Type of breeding site was a significant covariate in the GLMM. The model indicated that fishponds were 4.42 (95 % CI = 2.84–6.88) times more infested with anopheline larvae than natural breeding sites after correcting for repeated sampling (Table 3).

**Table 3:** Results from statistically significant models (*p* < 0.05) to assess larvae density in sampled natural and artificial water bodies in Mâncio Lima, Acre, Brazil between February 2012 and March 2014

Analysis	Dataset	Variable	Coefficient	Standard error	*P* value
1	D5	Water body type: fish pond	−1.486	0.219	<0.001
2	D3f	Vegetation	0.993	0.471	0.035
	D3n	Electrical conductivity	−0.046	0.013	<0.001
		Distance	−0.015	0.006	<0.01
3	D9f	Survey 2	−0.026	0.014	<0.001
		Survey 3	−1.626	0.013	<0.001
		Survey 4	−0.006	0.013	0.633
		Survey 5	0.077	0.013	<0.001
		Survey 6	−0.272	0.014	<0.001
		Survey 7	−0.193	0.013	<0.001
		Survey 8	−1.275	0.013	<0.001
		Survey 9	−0.055	0.013	<0.001
	D5n	Survey 3	0.999	0.650	0.1240
		Survey 5	1.093	0.609	0.0727
		Survey 7	0.096	0.721	0.8932
		Survey 9	−0.446	0.742	0.5476
5	D5f	Vegetation: non-commercial ponds	Smooth	–	0.614
		Vegetation: commercial ponds	Smooth	–	<0.001

### Influence of limnological and ecological factors on larval infestation

Larval abundance in fishponds was unaffected by water temperature, turbidity, dissolved oxygen, nitrate, ammonia, electrical conductivity, chlorophyll, pH, presence of macrophytes, and distance to the nearest house (*p* > 0.05). The proportion of border vegetation was the only significant covariate (*p* < 0.05) (Table 3). Every 10 % increase of vegetation (proportion of the breeding site border with presence of vegetation) caused a 10 % increase in larval abundance.

The percentage of border vegetation was not estimated for natural water bodies. Among the remaining variables, only electrical conductivity and distance to the nearest house were significant negative predictors in univariate models. The final model included both of these variables (Table 3).

### Temporal patterns of larval abundance

**Fig. 3:**
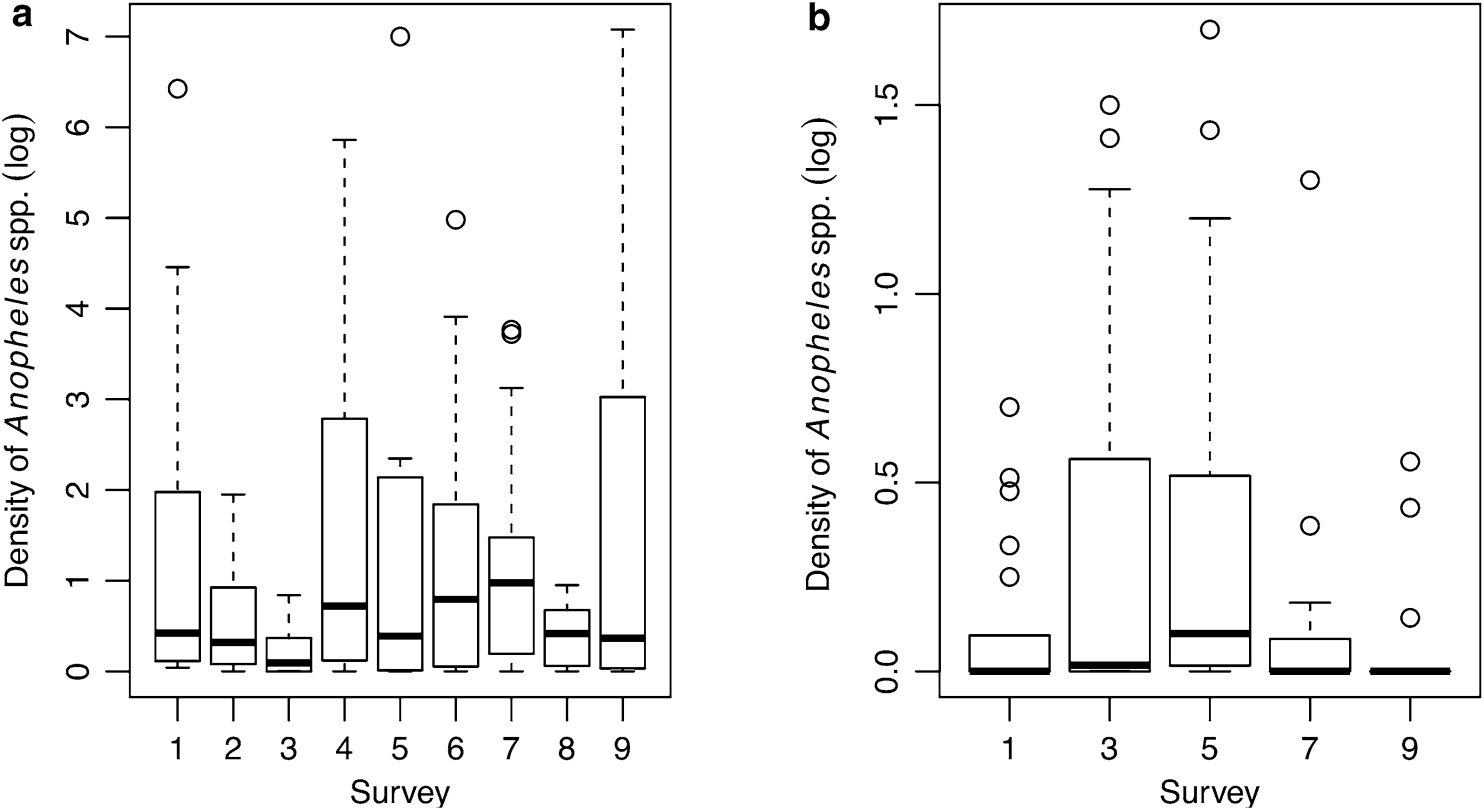
Temporal pattern of *Anopheles* spp. larval density (mean number of larvae per dip). **a** In fishponds sampled during each of the five complete and four fast surveys; **b** in natural water bodies surveyed only during the five complete surveys

The temporal abundance of anopheline larvae varied significantly between collections in fishponds (Fig. 3a; Table 3) but not in natural water bodies (Fig. 3b). Variation in larval abundance in both types of breeding sites was not associated with the season of collection (represented by the variable month).

### Effect of fishpond owner on larval infestation

**Fig. 4:**
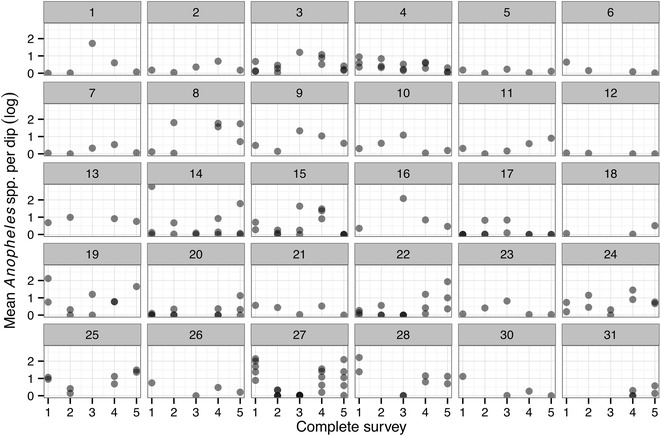
*Anopheles* spp. larval density (log-scale) in each fishpond and each complete survey by owner (*panel numbers* indicate individual owners). *Darker dots* indicate multiple overlapping fishponds

When comparing two models, one with the house owner ID and the other with water body ID as random effects, the model with owner ID was slightly better in terms of AIC values (AIC = 2233 and 2230, respectively). In other words, extent of infestation differed between owners, and ponds from the same owner exhibited similar degrees of infestation (Fig. 4).

Of all variables investigated in the questionnaire (commercial pond use, type of fish species bred in the pond, emptying, and type of fish food), the only variable that significantly influenced larval density was whether or not the pond was used for commercial use. Fishponds that contained fish intended for sale (40 of 55) were 1.7 times more infested (estimated effect size = 0.52, *p* = 0.037) than those with fish for family consumption.

**Fig. 5:**
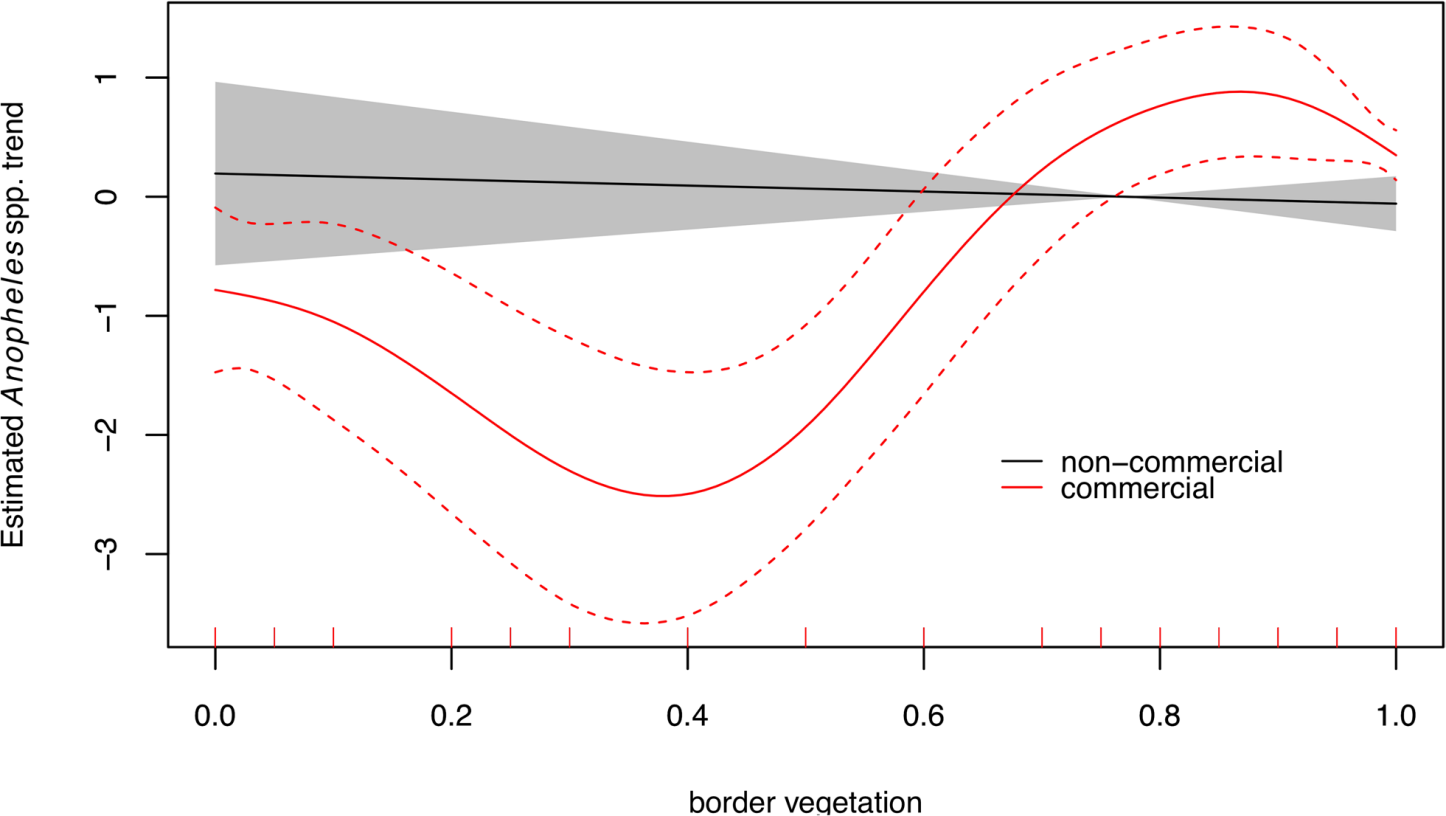
Result of a generalized additive mixed model (GAMM) incorporating a smoothing effect for border vegetation in commercial and non-commercial fishponds

Figure 5 shows results from models with smoothing functions for border vegetation in both commercial and non-commercial fishponds. The model indicates that (1) larval infestation in non-commercial fishponds was not affected by the proportion of the border covered by vegetation; (2) commercial ponds were less infested with larvae than non-commercial ponds when less than 65 % of the border had vegetation; and, (3) when border vegetation exceeded 65 %, commercial ponds were more infested than non-commercial ponds (Table 3).

## Discussion

The aquatic phase of immature mosquitoes is a critical stage in the mosquito life cycle. The distribution and abundance of many disease vector species, including malarial vectors, is directly related to the characteristics of the vector’s breeding sites [10]. Both natural water bodies and fishponds were infested with anopheline larvae in this Amazonian malaria-endemic town. Fishponds however were on average four times more infested with anopheline larvae than natural water bodies. These results support the findings from a previous study from 2008 in Manaus, Brazil, where fishponds were also four times more infested than other available mosquito breeding sites [26]. Fishponds were also identified as breeding sites for *An. darlingi* in the northeastern Peruvian Amazon [27], where presence of fishponds were risk factors for human malaria transmission [28]. Fishponds become suitable habitats for *An. darlingi*, the most important malaria vector [1] as it adapts to man-made breeding habitats, preferring deep, stable, clear water bodies in proximity to human dwellings [1, 28], despite potential larval predation by juvenile fish [27, 28].

The high larval abundance in fishponds suggests that fish predation in these habitats is not completely sufficient to prevent larval development. It was hard to ascertain the specific fish community of each pond, but personal observations indicate that most ponds had a mixed community of native and introduced species, combining diverse species, such as Nile tilapia (*Oreochromis niloticus)*, pacu (*Piaractus brachypomus*), curimatã *(Prochilodus* spp.), tambaqui (*Colossoma macropomum)*, spotted sorubim (*Pseudoplatystoma corruscans*), and piauaçu (*Leporinus macrocephalus*). Of these species, *Oreochromis niloticus* has been used for anopheline larval control in Africa [29]. While some studies show that the introduction of tilapia and mosquitofish (*Gambusia affinis*) into active fishponds have drastically reduced the density of *Anopheles gambiae* and other anophelines in Africa [30, 31], other studies show no such effect [32, 33]. The predatory effect of fishes can be reduced by other factors such as (1) the presence of a dense border vegetation and floating plant parts (offers hiding places for the mosquito larvae, which reduces the efficiency of larvivourous fish); (2) insufficient number of fishes in the ponds; (3) high larval productivity (due to a large adult mosquito population); (4) growth stage of the fish (only fingerlings feed preferentially on mosquito larvae); and, (5) feeding behaviour of the bred fish species (curimatã and spotted sorubim mainly feed on the ground and only rarely on the water surface [34–37]).

Another factor that might have influenced the survival of anopheline larvae in fishponds is a likely different fauna of predatory aquatic insects in the two types of water bodies. An inspection of the insects found in the dips suggests a variety of species that could act as larva predators in fishponds: Odonatas, Hemiptera, Coleoptera, and Diptera. Further studies should investigate the impact of these species on larval dynamics in natural water collections and fishponds.

Strong economic incentives for the development of fish farming in the State of Acre, especially in the Juruá region, where Mâncio Lima is located, have caused rapid changes in the urban landscape, and a direct impact on the risk of malaria infection in the region. A total of 14,310 malaria cases were notified in Mâncio Lima during the study period (13,387 from which 94 % were autochthonous). A previous work of our group already showed that fishpond construction effort (2003–2006) coincided both spatially and temporally with the increased number of malaria notifications [15]. These findings reinforce the association between the reproductive behaviour of malaria vectors and this economic activity [27].

Anopheline density was higher in natural water bodies that were close to households. This suggests an elevated exposure of these residents to the malaria vector in comparison to residents living further away. A direct association between malaria prevalence and *An. darlingi* larvae abundance in fishponds at distances of less than 100 m in Mâncio Lima was already observed [15]. It was previously shown that an increasing distance between humans and breeding sites reduces the contact between humans and anthropophilic malaria-vector mosquitoes, such as *An. darling*i, and therefore the risk of malaria infection [38].

Previous studies suggest that abiotic factors (pH, temperature, nitrate, ammonia, sulfate, turbidity, electrical conductivity, and chlorophyll) as well as biotic factors (vegetation, predation and competition) affect the development and survival of anopheline larvae [39–42]. However, the physical–chemical characteristics of breeding sites does not often explain the preference of anophelines for certain habitats [43, 44].

In the present study, pH, temperature, ammonium, chlorophyll, nitrate, dissolved oxygen, and turbidity were not associated with the occurrence of *Anopheles* spp. larvae in both artificial and natural breeding sites. Some reasons for this result include low measurement variation between water bodies (temperature and pH, for example) and lack of statistical power (small effect compared to the sample size because limnological data were only collected during the first three complete surveys). A study from western Kenya also did not detect any significant association between the occurrence of *An. gambiae* larvae and several habitat variables [45]; McKeon et al. [10] showed that water temperature was not associated with the occurrence of *Culex* and *Anopheles*.

The only significant predictor for the number of anopheline larvae in fishponds was the per cent of border with vegetation (in a positive relationship). Anopheline larvae use border vegetation to hide from potential predators [39]. Studies in Peru, Venezuela and Colombia indicated that emerging water body vegetation (especially Graminae) were risk factors for the presence and maintenance of *Anopheles* spp. [27, 46, 47]. It is interesting to note that the effect of border vegetation in Mâncio Lima was different in commercial and non-commercial fishponds. *Anopheles* spp. infestation of commercial fishponds was associated with borders that had more than 65 % vegetation cover. Considering that commercial fishponds have more fish than those for family use, this suggests an interaction between fish predation and vegetation border. Fishes can control larvae in fishponds with up to 65 % vegetation cover. Beyond that, the extent of the hiding space for larvae is sufficient to counteract the effects of predation. This result points to an objective target for pond management but further studies should investigate its effect on a controlled experimental design. Larval infestation in non-commercial fishponds was not influenced by the presence or absence of border vegetation. This difference between commercial and non-commercial fishponds might be due to differences in the fish species and abundance, a hypothesis for future investigations.

In the natural water bodies, on the other hand, an inverse association between anopheline larval abundance and water electrical conductivity was found, which reinforces previous results from West Africa and the Brazilian Amazon [10, 11, 48]. Conductivity increases when ions are liberated through the decomposition process, and is an indirect measure of water`s pollutant concentration [49]. It is known that anopheline species are sensitive to pollution. Larval infestation in fishponds and natural water bodies did not change significantly between the seasons. For the fishponds this is probably due to the fact that they are unaffected by seasonal changing water levels [35]. Natural water bodies were most likely unaffected by the season, because larval density was extremely low in comparison to fishponds. This was different in several collection points in the Azul River in Boa Vista (Roraima, Brazil), where *An. darlingi* larvae were absent during the rainy season, and present in the dry season [38]. Vittor et al. [27], on the other hand, found a moderate positive association of the presence of *An. darlingi* larvae with the rainy season.

Infestation levels differed between fishponds, and ponds from the same owner tended to have similar extents of infestation. Fishponds owned by the same farmer could exhibit similar degrees of infestation because of their spatial aggregation, fish life cycle, species of fish, fish feeding schedule, and distance between the pond and the edge of the forest. These variables were not included in the present study, yet other studies have described an association between each of them and subsequent anopheline breeding and malaria transmission [21, 26, 35].

## Conclusion

The findings reinforce the importance of developing and implementing feasible good practice programmes for both commercial and non-commercial fish farmers in order to reduce the risk of malaria in their families and communities. Good practices should include protocols for inspection and removal of excess vegetation from the borders as well as floating vegetation on the surface of their ponds, regulation of water level [39], and appropriate use of herbicides or algicides. Furthermore, biological larvicides, such as *Bacillus sphaericus* (Bs) or *Bacillus thuringiensis* var. *israelensis* (Bti) could be considered as a control alternative in fishponds [26, 32]. It is also recommended to evaluate if a combination of fish species could enhance their predatory effect on anopheline larvae, providing a biological control alternative for this problem.

Importantly, it must be acknowledged that any of these tasks can be daunting for those working alone or with low resources. There are several barriers to good fishpond management practices, including but not limited to a general lack of knowledge, proper equipment, financial resources, or time. Identifying and elucidating the influence of each of these barriers is important for the development of effective interventions. Especially, an aquaculture malaria control programme should consider a collaborative approach to help low-resource farmers, for example, stimulating their participation and organization of cooperatives.

The present study suggests that fishponds serve as important and productive breeding sites for malaria vectors. They therefore contribute to the ongoing transmission of malaria in the Brazilian Amazon. Adequate fishpond maintenance must be promoted with the aim of rendering fishponds less desirable for anopheline larvae, most importantly *An. darlingi* in malaria-endemic regions.
